# Telomere heterogeneity linked to metabolism and pluripotency state revealed by simultaneous analysis of telomere length and RNA-seq in the same human embryonic stem cell

**DOI:** 10.1186/s12915-017-0453-8

**Published:** 2017-12-08

**Authors:** Hua Wang, Kunshan Zhang, Yifei Liu, Yudong Fu, Shan Gao, Peng Gong, Haiying Wang, Zhongcheng Zhou, Ming Zeng, Zhenfeng Wu, Yu Sun, Tong Chen, Siguang Li, Lin Liu

**Affiliations:** 1 State Key Laboratory of Medicinal Chemical Biology, Nankai University, Tianjin, 300071 China; 2 Department of Cell Biology and Genetics, College of Life Sciences, Nankai University, Tianjin, 300071 China; 3 Stem Cell Translational Research Center, Tongji Hospital, Tongji University School of Medicine, Shanghai, 200065 China; 4 Department of Obstetrics, Gynecology and Reproductive Sciences, Yale School of Medicine, New Haven, CT 06511 USA; 5 EHBIO Gene Technology co., LTD, Beijing, 100029 China

**Keywords:** Single cell analysis, Telomere length, Transcriptome, Human embryonic stem cell, Heterogeneity

## Abstract

**Background:**

Telomere length heterogeneity has been detected in various cell types, including stem cells and cancer cells. Cell heterogeneity in pluripotent stem cells, such as embryonic stem cells (ESCs), is of particular interest; however, the implication and mechanisms underlying the heterogeneity remain to be understood. Single-cell analysis technology has recently been developed and effectively employed to investigate cell heterogeneity. Yet, methods that can simultaneously measure telomere length and analyze the global transcriptome in the same cell have not been available until now.

**Results:**

We have established a robust method that can simultaneously measure telomere length coupled with RNA-sequencing analysis (scT&R-seq) in the same human ESC (hESC). Using this method, we show that telomere length varies with pluripotency state. Compared to those with long telomere, hESCs with short telomeres exhibit the lowest expressions of *TERF1*/*TRF1*, and *ZFP42/REX1*, *PRDM14* and *NANOG* markers for pluripotency, suggesting that these hESCs are prone to exit from the pluripotent state. Interestingly, hESCs ubiquitously express *NOP10* and *DKC1*, stabilizing components of telomerase complexes. Moreover, new candidate genes, such as *MELK*, *MSH6*, and *UBQLN1*, are highly expressed in the cluster of cells with long telomeres and higher expression of known pluripotency markers. Notably, short telomere hESCs exhibit higher oxidative phosphorylation primed for lineage differentiation, whereas long telomere hESCs show elevated glycolysis, another key feature for pluripotency.

**Conclusions:**

Telomere length is a marker of the metabolic activity and pluripotency state of individual hESCs. Single cell analysis of telomeres and RNA-sequencing can be exploited to further understand the molecular mechanisms of telomere heterogeneity.

**Electronic supplementary material:**

The online version of this article (doi:10.1186/s12915-017-0453-8) contains supplementary material, which is available to authorized users.

## Background

Telomeres are the highly repetitive ribonucleoprotein structures (TTAGGG)_n_ that protect chromosome ends and maintain genomic instability [1, 2]. Short telomeres are associated with aging and tumorigenesis [3–5]. Yet, tumors, and particularly those positive for alternative lengthening of telomeres (ALT), are characterized by remarkable telomere length heterogeneity [6]. Notably, functional telomeres are required for embryo development and for the pluripotency and differentiation capacity of embryonic stem cells (ESCs) [7, 8]. Pluripotent stem cells, including ESCs, induced pluripotent stem cells (iPSCs), and nuclear transfer ESCs with sufficient telomere lengths, are able to give rise to offspring shown by germline chimera production or tetraploid embryo complementation tests, the most stringent and functional test of naïve pluripotency [7, 9–11]. While short telomeres impair stem cell differentiation [12], ESCs with hyper-long telomeres may delay aging as evidenced by the generation of healthier chimera mice that exhibit reduced cell senescence and DNA damage with age and better skin wound healing [13].

Telomeres are elongated during early expansion of human ESCs (hESCs) and then reach a relatively stable length [14]. Variations in telomere lengths are found in human iPSCs [15, 16]. Additionally, ESC cultures exhibit heterogeneous expression of transcription factors of naïve pluripotency such as *NANOG* [17, 18], *REX1/ZFP42* [19, 20], *STELLA*/*DPPA3* [17, 18], *ESSRB* [17, 19], *KLF4* [19, 21], and *TBX3* [17, 18]. Unlike mouse ESCs, functional tests of pluripotency of hESCs are limited due to ethics issues [22]. It remains elusive how telomere lengths and heterogeneity are implicated in pluripotency and differentiation of human ESCs/iPSCs.

Conventional methods for the measurement of telomere lengths include telomere restriction fragment (TRF) [23], Q-FISH (quantitative fluorescence in situ hybridization) [24], Flow-FISH (flow cytometry method using fluorescence in situ hybridization) [25], T-OLA (telomeric-oligonucleotide ligation assays) [26], qPCR (real-time quantitative polymerase chain reaction) [27], and STELA (single telomere length analysis) [28]. TRF measurement requires large amounts of DNA (micrograms) and consistently produces large telomere DNA fragment smears, which itself may indicate telomere length heterogeneity. Conventional qPCR does not need large amounts of DNA (nanogram), but still requires a cell population rather than a single cell. Q-FISH is normally used to estimate telomere lengths of metaphase chromosome spread prepared from a cell population.

Telomeres may be influenced by gene mutations shown by genomic sequencing analysis in a large cell population or complex tissues [29, 30]. The bulk cell population in the same environment also displays cell-to-cell variations in gene expression [31]. Single-cell genome sequencing (DNA-seq and RNA-seq) has rapidly developed into a powerful method to investigate genetic and transcriptome heterogeneity in a cell population [32–34]. By combining methods to amplify the telomere-specific sequences and mRNA/cDNA separately, we were able to simultaneously measure telomere length coupled with RNA-sequencing analysis in the same cell, and employed this method to investigate telomere length heterogeneity and pluripotency of hESCs.

## Results

### Simultaneous measurement of telomere length and gene expression in the same cell

**Fig. 1:**
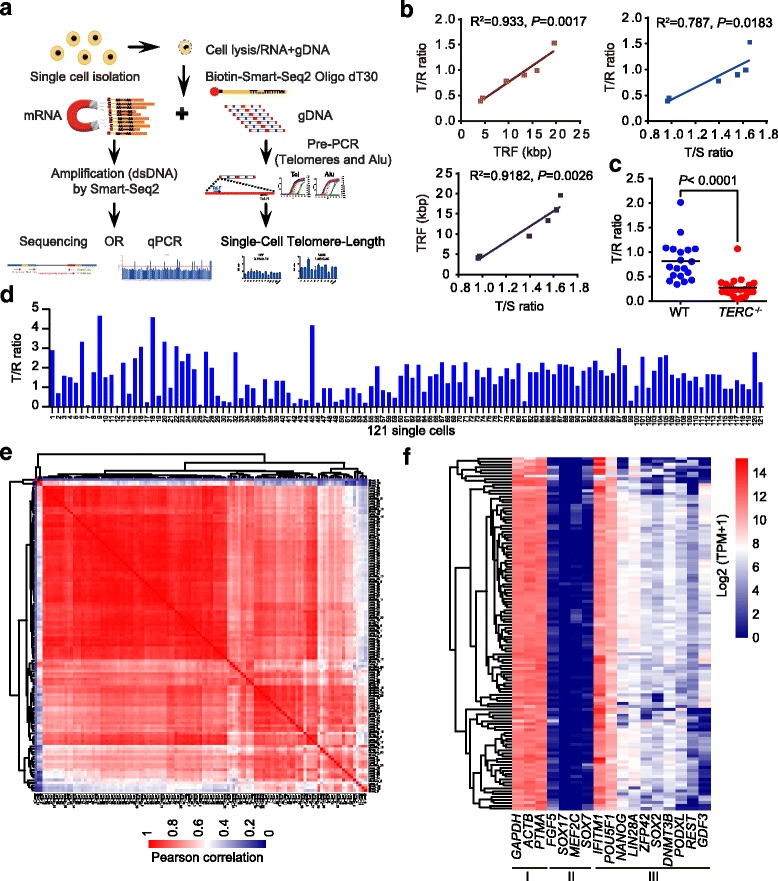
Simultaneous measurement of telomere length and transcriptome in the same cell. **a** Overview and design of single-cell telomere length measurement and RNA-sequencing analysis (scT&R-seq) in the same cell. **b** Average telomere length in single cells shown as T/R ratio by scT&R-seq is significantly correlated with that of other methods in various human cell lines. Each point represents one cell line, and different colors distinguish between methods. **c** Relative telomere length of *TERC* knockout and wild-type hESCs using scT&R-seq. **d** Relative telomere length distribution of all individual human embryonic stem cell (hESC, WA26). **e** Hierarchical clustering analysis of whole-gene expression similarity (measured by Pearson correlation coefficients) for all hESCs. **f** Heat-map showing expression of selected marker genes for housekeeping (I), differentiation (II), and pluripotency (III) in hESCs. Each row represents one cell

To acquire the total mRNA and genomic DNA (gDNA) from the same cell, we used the biotinylated oligo-dT primer [35], targeting to mRNA containing a polyadenylated tail (poly (A)^+^) and applied a magnet to separate the gDNA from the same cell (Fig. 1a). We named this novel method as single cell telomere length and transcriptome sequencing (scT&R-seq; see Methods).

To validate the feasibility of the method, we analyzed telomere length and relative gene expression in single U2OS (osteosarcoma cells) and hESCs (WA26) (Additional file 1: Figure S1a–c). Following pre-amplification specific for telomeres (T) and multicopy repeat reference gene *Alu* (R) of gDNA in parallel, the Ct value for *Tel* and *Alu* in negative controls were much greater than those of single cells by qPCR (Additional file 1: Figure S1a). Following reverse transcription of mRNA and amplification of cDNA, the variation in the expression of *GAPDH* by qPCR, a housekeeping gene, was minimal. The pluripotency gene *POU5F1*/*OCT4* was only highly expressed in WA26 but not in U2OS, as expected. No product was detected in the negative controls (Additional file 1: Figure S1c).

Because this new method involves physical separation of DNA from mRNA, we tested whether the separation step influenced the telomere length detection at single cell level from the same cell line (WA26). Twenty cells were randomly picked and subject to single cell telomere length analysis using single-cell telomere length measurement by qPCR, without a separation step [36], or scT&R-seq (Additional file 1: Figure S1d). Ct values of *Tel* (*P* = 0.1879) and *Alu* (*P* = 0.1044), as well as telomere length, did not differ (*P* = 0.6525) in single cells measured by the methods regardless of the separation technique (Additional file 1: Figure S1d). We further validated single-cell telomere length measurement by TRF (in kb) and conventional qPCR (T/S ratio) in hESCs (WA26, RuES2), U2OS, human colorectal cancer cells (HCT116), cervical cancer cells (HeLa S3), and human embryonic fibroblast (HEF) cells (Additional file 1: Figure S1e–h). The average relative telomere length shown as T/R ratio (Additional file 1: Figure S1e) of single cells measured by scT&R-seq was highly correlated with that measured by the two conventional methods applied to a cell population (Fig. 1b). Further, the T/S ratio also correlated well with TRF in all six cell lines tested (Fig. 1b). Variations in telomere length revealed by scT&R-seq were readily detected by TRF (Additional file 1: Figure S1h). Remarkably, telomere lengths differed among single cells within the same cell population. hESCs (WA26) were observed to normally express POU5F1/OCT4 by fluorescence microscopy and telomerase activity was observed by Telomeric Repeat Amplification Protocol (TRAP) assay (Additional file 1: Figure S1i, j).

To further validate the reliability of scT&R-seq, we measured telomere length by both Southern blot and scT&R-seq methods of telomerase gene-knockout hESCs after deletion of *TERC* by CRISPR/Cas9 and compared these with wild-type (WT) controls (Additional file 2: Figure S2a). Compared to WT hESCs, telomerase-deficient (*TERC* ^-/-^) hESCs showed decreased telomerase activity by TRAP assay (Additional file 2: Figure S2b), and generally shorter telomeres measured by TRF (Additional file 2: Figure S2c), as expected. Telomere length measured at single cell level by scT&R-seq was notably shorter (*P* < 0.0001) in *TERC* ^-/-^ than in WT hESCs (Fig. 1c; Additional file 2: Figure S2d). While relative expression levels shown as Ct values of housekeeping gene *GAPDH* did not differ (*P* = 0.0726) between *TERC* ^-/-^ and WT hESCs (Additional file 2: Figure S2e), the expression level of *NANOG* was reduced at the single cell level in *TERC* ^-/-^ compared with that of WT hESCs (Additional file 2: Figure S2f), consistent with the bulk results (Additional file 2: Figure S2g). Further, the expression level of *NANOG* was correlated with telomere length, shown as T/R ratio (Additional file 2: Figure S2h).

### Single-cell analysis of telomere length and global transcriptome in hESCs by scT&R-seq

To investigate the potential relationship in heterogeneity of transcriptome and telomere length, 121 single hESCs were randomly selected for single cell analysis using scT&R-seq. Both telomere length and transcriptome displayed high heterogeneity (Fig. 1d, e, Additional file 3: Table S1). Telomere length of single cells ranged from 0.04 to 4.64 (Fig. 1d). The correlation coefficient of the global gene expression profile varied from 0.1 to 1.0 (Fig. 1e), revealing the heterogeneity of hESCs at global transcriptome levels. Housekeeping (class I) and pluripotency-related transcripts (class III) at single cell level were highly expressed in hESCs, and differentiation-related genes (class II, e.g., *SOX7*, *MEF2C*, *SOX17*, *FGF5*) were maintained at relatively lower expression levels (Fig. 1f).

All single cell RNA-seq libraries showed remarkably uniform gene body coverage profiles from the 5′ to 3′ end of each gene (Additional file 4: Figure S3a). Most of the expressed genes were detected even with down-sampling of 50% of the reads (Additional file 4: Figure S3a). The sequencing depth of all individual libraries was enough for a subsequent reliable analysis based on previous reports [37]. All single cell RNA-seq data were used for downstream analyses. The RNA-seq libraries with an average of 3.3 million single cell RNA-seq reads per cell were obtained and the average ratio mapped was 69.65% (Additional file 4: Figure S3b, c). Most of the detected genes had transcripts per million values (TPM) of over 10, and 26,321 genes were identified when all single cell libraries were combined (Additional file 4: Figure S3d, Additional file 5: Table S2). Of these, the top 40 genes expressed in single cells included housekeeping genes, ribosomal protein genes, and pluripotency genes (e.g., *POU5F1*/*OCT4* and *IFITM1*). In addition, *TMSB4X*, *FTL*, *GSTP1*, *H2AFZ*, *SLIRP*, and *UBB* had highly expressed abundance in hESCs (Additional file 4: Figure S3e). A strong correlation was found between single-cell RNA-seq and qPCR for *TERF1* and *POU5F1*/*OCT4* (Additional file 6: Figure S4), further validating the single cell RNA-seq data. Cell cycle stage may have influenced the heterogeneity of gene expression [38]. We employed the prediction method (single-cell latent variable model, scLVM) to classify cells into cell cycle phases based on gene expression data. Only 11.6% of variation was attributable to the cell cycle phases, and cell cycle stage had minimal effects on the clustering results. No obvious differences in telomere length were found at different cell cycle stages (Additional file 4: Figure S3f).

### Telomere length heterogeneity linked to specific gene expression profile

**Fig. 2:**
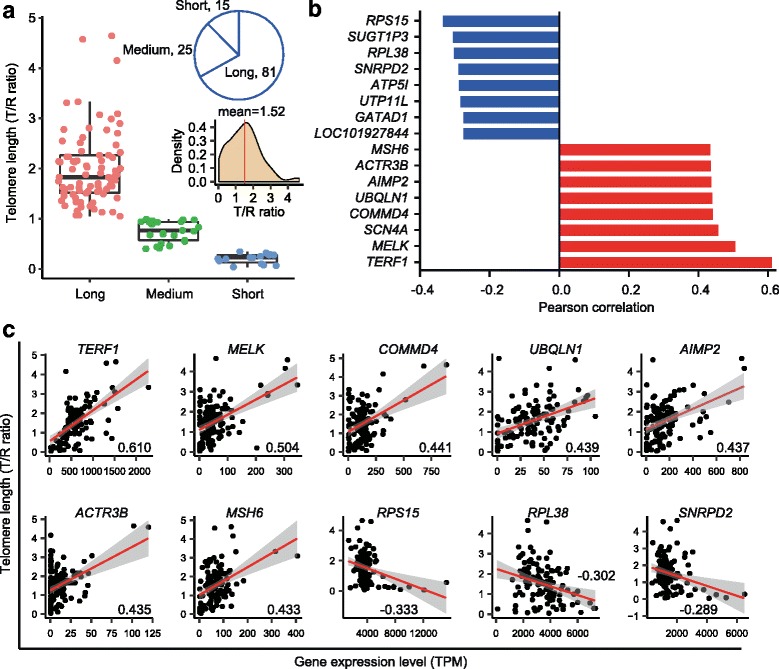
Telomere length heterogeneity and candidate genes potentially involved in telomere length maintenance of human embryonic stem cells (hESCs). **a** hESCs were clustered into three telomere length groups based on T/R ratio by single-cell analysis. We defined the cells with T/R ratio over 1.0 as the “long” telomere group, those with T/R ratio 0.4–1.0 as the “medium” group, and those with T/R ratio under 0.4 as the “short” group. Inset pie figure shows cell number in each telomere length group. Inset density figure indicates distribution of telomere length of hESCs. **b** Top eight genes with expression patterns remarkably correlated with telomere length (T/R ratio). Red and blue bars show positive and negative correlation with each other. **c** Scatter plots showing correlation between gene expression level and T/R ratio for selected genes in all samples. The line represents fitted values and shaded band shows 95% confident regions. Correlation value of each gene is shown on the plot

Telomere length (T/R ratio) distribution in hESCs varied, with a mean value of 1.52. Therefore, we used cutoffs for the three telomere length groups based on the T/R ratio (single cell telomere length) (see Methods). hESCs and telomere ALT positive cells (U2OS) showed longer telomere lengths (long group), and differentiated HEF cells had shorter telomeres (short group) (Fig. 2a, Additional file 3: Table S1). Eighty-one cells showed a T/R ratio centered from 1.00 to 4.64 (long group), and fewer hESCs (25 and 15, respectively) exhibited medium or short telomeres (inset figures in Fig. 2a).

By searching for the relationship of telomere lengths and gene expression levels, we identified the candidate genes that might be directly or indirectly involved in telomere length regulation according to the top positive and negative correlation coefficient (Fig. 2b, Additional file 7: Table S3, Additional file 8: Table S4). Expression of *TERF1*, a key component factor of telomere shelterin complex important for telomere maintenance [39], was highly and positively correlated with telomere length (Fig. 2c). Expression levels of DNA mismatch repair protein *MSH6* were also highly associated with hESC telomere lengths. Others, including *MELK*, *SCN4A*, *COMMD4*, *UBQLN1*, *AIMP2*, and *ACTR3B*, also showed a positive correlation with telomere length (Fig. 2c). In contrast, *RPS15*, *SUGT1P3*, *RPL38*, *SNRPD2*, *ATP5I*, *UTP11L*, *GATAD1*, and *LOC101927844* were negatively correlated with telomere length (Fig. 2c).

**Fig. 3:**
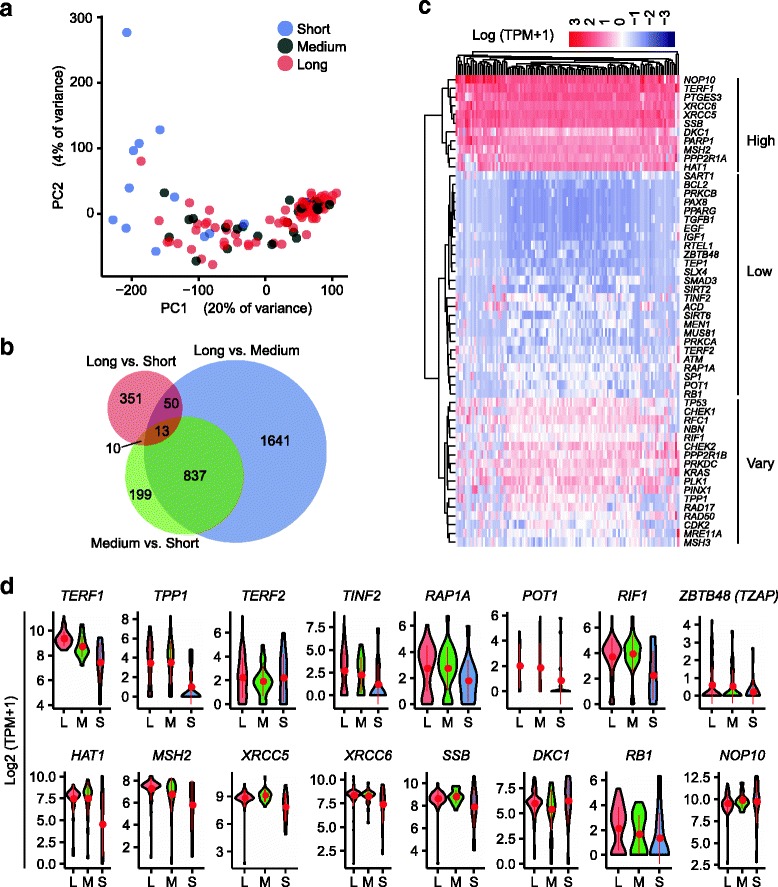
Classification of telomere lengths in association with global transcriptome of human embryonic stem cell (hESCs). **a** Classification of all single cells based on whole transcriptome profile using principal component analysis (PCA, scatter plot of component 1 and 2), with cells colored according to telomere length group. **b** Venn diagram depicting the overlap among three telomere groups. **c** Heatmap representing expression levels of 54 selected genes related to telomere length maintenance from GESA gene sets (http://software.broadinstitute.org/gsea). “High” means gene expression at high levels; “Low” means low expression levels; “Vary” indicates gene expression levels in a wide range. **d** Violin plots showing relative expression levels of genes associated with shelterin complex in each telomere length group (*L* long, *M* medium, *S* short). Y-axis indicates Log2 (TPM + 1)

To explore the molecular identities of individual cells by combination of telomere length and gene expression, we performed global principal component analysis (PCA) projection of all single cells. Long or short telomere hESCs could be mostly separated by clustering of PC1 and PC2 (Fig. 3a, Additional file 9: Table S5). To exclude the possibility that the cluster might be caused by confounder and artifact of low mapping ratios, we employed the *scater* package (single-cell analysis toolkit for expression in R) to compute the cumulative proportion of reads [40]. Cells from these three telomere length groups displayed the same cumulative proportion of reads (Additional file 10: Figure S5a). The mapping ratio of raw reads showed no significant correlation (*P* = 0.3884) with single cell telomere length (Additional file 10: Figure S5b), indicating that the result of PCA was not an artifact.

Analysis of differentially expressed genes between long, medium, or short telomere groups showed that 13 genes were commonly shared among these three separate comparisons (long vs. short, long vs. medium, medium vs. short) (Fig. 3b, Additional file 11: Table S7). The 351 genes were differentially expressed in long telomere compared with short telomere cells, and 50 genes were shared in long versus short and medium versus short telomere cells (Fig. 3b). We performed Gene Ontology (GO) and Kyoto Encyclopedia of Genes and Genomes (KEGG) pathway enrichment analyses for the differentially expressed genes in long versus short telomere cells. The highest enrichment was linked to neuroactive ligand-receptor interaction, extracellular matrix-receptor interaction of KEGG pathway, and the metabolic biological process in long telomere cells, relative to short telomere cells (Additional file 12: Figure S6).

By hierarchical clustering [41] of telomere maintenance genes from GSEA (Gene Set Enrichment Analysis) (Additional file 13: Table S6), we observed that *NOP10*, *DKC1*, *TERF1*, *PTGES3*, *XRCC5*, *XRCC6*, and *SSB* were expressed at high levels in almost all hESCs (Fig. 3c); *NOP10* and *DKC1* are two components known as stabilizers of the telomerase complex [42]. Analysis of six shelterin complex proteins *TERF1*, *TERF2*, *TINF2*, *TPP1*, *RAP1A*, and *POT1* [39] in different telomere length groups revealed that *TERF1* was consistently highly expressed in long telomere hESCs and less expressed in short telomere cells (Fig. 3d). *TPP1* and *RAP1A* were also expressed at higher levels in long and medium telomere hESCs than in short telomere cells (Fig. 3d). *TZAP* (also named *ZBTB48*) was recently identified as a new telomere-associated gene involved in telomere length control [43]; hESCs generally expressed *TZAP* at a low level, and rarely at a high expression level (Fig. 3d). Of other genes potentially expressed for telomere maintenance, *HAT1*, *MSH2*, *XRCC5*, *XRCC6*, and *SSB* were expressed at lower levels in short telomere hESCs (Fig. 3d).

### Telomere length associated with pluripotency

**Fig. 4:**
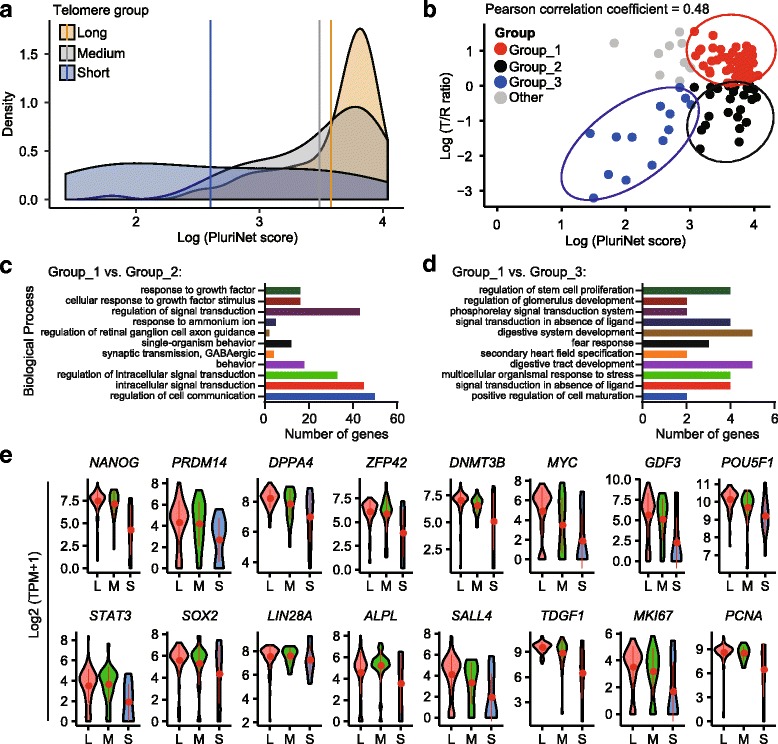
scT&R-seq analysis of heterogeneity in pluripotency gene expression of hESCs. **a** Distribution of PluriNet score. Gene set for PluriNet score was generated from study in GEO (Chu et al*.* [45]). **b** Correlation between the averaged PluriNet score and telomere length (T/R ratio). hESCs were divided into three groups by Log (PluriNet score) and Log (T/R ratio), Group_1 (Log (T/R ratio) > 0; Log (PluriNet score) > 3.0), Group_2 (Log (T/R ratio) ≥ 0; Log (PluriNet score) ≥ 3.0), and Group_3 (Log (T/R ratio) < 0; Log (PluriNet score) < 3.0). **c** Functional annotation of genes showing higher expression in Group_1 compared to Group_2. **d** Functional annotation of genes with higher expression in Group_1 compared to Group_3. **e** Violin plots detailing single cell telomere length distribution and expression of pluripotency genes in each telomere length (*L* long, *M* medium, *S* short) group. Y-axis indicates Log2 (TPM + 1)

To investigate whether telomere lengths correlated with expression levels of pluripotent genes, we defined a pluripotency score of hESCs by average expression of 299 pluripotent genes from PluriNet [44]. We validated the pluripotency score (named PluriNet score) using the single cell data obtained from human pluripotent stem cells and differentiated cells reported by Chu et al. [45]. As expected, the PluriNet score was higher in undifferentiated hESCs than in differentiated cells from the same precursor cell (Additional file 14: Figure S7a). We used this score to define the pluripotency of hESCs with various telomere lengths. Short telomere hESCs displayed a lower PluriNet score compared with hESCs in the long and medium telomere groups (Fig. 4a, Additional file 14: Figure S7b). Pearson’s correlation analysis of telomere length (Log(T/R ratio)) and Log(PluriNet score) showed a positive association of the two factors with a coefficient of approximately 0.48 (Fig. 4b, Additional file 3: Table S1). We divided hESCs into three groups, Group 1 representing high pluripotency with long telomere length, Group 2 representing high pluripotency with short telomere length, and Group 3 representing low pluripotency with short telomere length (Fig. 4b).

GO biological process enrichment indicates that the genes highly expressed in Group 1 compared with Group 2 involved regulation of cell communication, cellular response to growth factor stimulus, and response to growth factor (Fig. 4c, Additional file 15: Table S8). Genes highly expressed in Group 1 compared with Group 3 involved regulation of stem cell proliferation (Fig. 4d).

Further analysis of 16 selected genes related to pluripotency showed that genes for pluripotency, *NANOG*, *PRDM14*, and *ZFP42/REX1* [17, 18], were expressed at lower levels in hESCs with short telomeres (Fig. 4e). *DNMT3B*, *MYC*, and *GDF3* were expressed at higher levels in general in long and medium telomere hESCs than in short telomere hESCs (Fig. 4e), consistent with their potential roles in maintaining the pluripotent state of human ESCs [17, 46, 47]. However, the relative expression level of each gene still varied among hESCs with long and medium telomere hESCs, and whether this relates to a naïve or primed state remains unclear. *POU5F1*/*OCT4*, *SOX2*, and *LIN28A* did not differ in their expression levels regardless of telomere lengths (Fig. 4e). *LIN28A* is expressed at markedly reduced levels in naïve versus primed states [48]. In contrast, cells with short telomeres expressed the genes primed for differentiation at higher levels, e.g., *FGF5* (Additional file 16: Figure S8). Thus, telomere length can mark the pluripotency state of hESCs.

### Distinct metabolic pathway in association with telomere length heterogeneity of hESCs

**Fig. 5:**
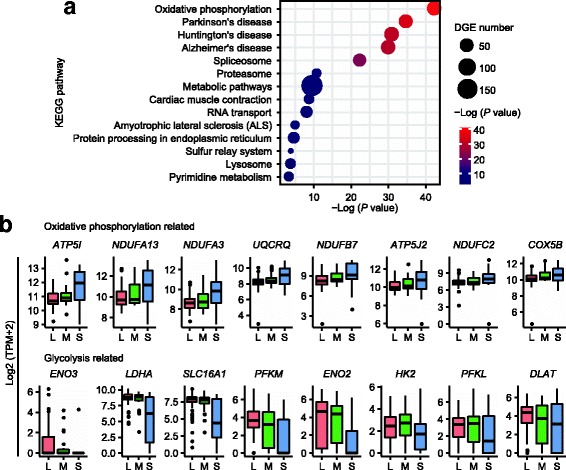
Short telomere human embryonic stem cells (hESCs) display increased oxidative phosphorylation and decreased glycolysis. **a** Top 14 KEGG pathway annotations of significantly up-regulated genes in short telomere group compared with long telomere group. The terms are sorted by their enrichment *P* value and the shape indicates the number of differentially expressed genes. **b** Boxplots illustrating distribution in the expression of genes associated with oxidative phosphorylation and glycolysis in the various telomere length (*L* long, *M* medium, *S* short) groups. Gene list of oxidative phosphorylation from KEGG and genes related to glycolysis were obtained from Gu et al. [49]

We performed KEGG pathway enrichment analysis of the differentially expressed genes of Group 1 compared with Group 3 (Fig. 5a, Additional file 17: Table S9). Specifically, genes involved in the oxidative phosphorylation metabolic pathway were more enriched in hESCs with short telomeres than in those of long telomeres (Fig. 5b). Genes associated with oxidative phosphorylation included *ATP5I*, *NDUFA13*, *NDUFA3*, *UQCRQ*, *NDUFB7*, and *ATP5J2* (Fig. 5b). In contrast, genes related to glycolysis metabolism *ENO3*, *SLC16A1*, *PFKM*, and *LDHA* [49] were up-regulated in hESCs with long telomeres, compared with those of short telomeres (Fig. 5b).

Pluripotent ESCs are metabolically characterized with high glycolysis activity, whereas primed ESCs and differentiated cells have an increased and even higher oxidative phosphorylation activity, respectively [49]. Our data support the idea that hESCs with short telomeres may tend to rely on the oxidative phosphorylation pathway in metabolism, further suggesting the reduced pluripotency or tendency to differentiate.

### Validation of *NANOG* and *TERF1* expression related to telomere length heterogeneity and pluripotency of hESCs

**Fig. 6:**
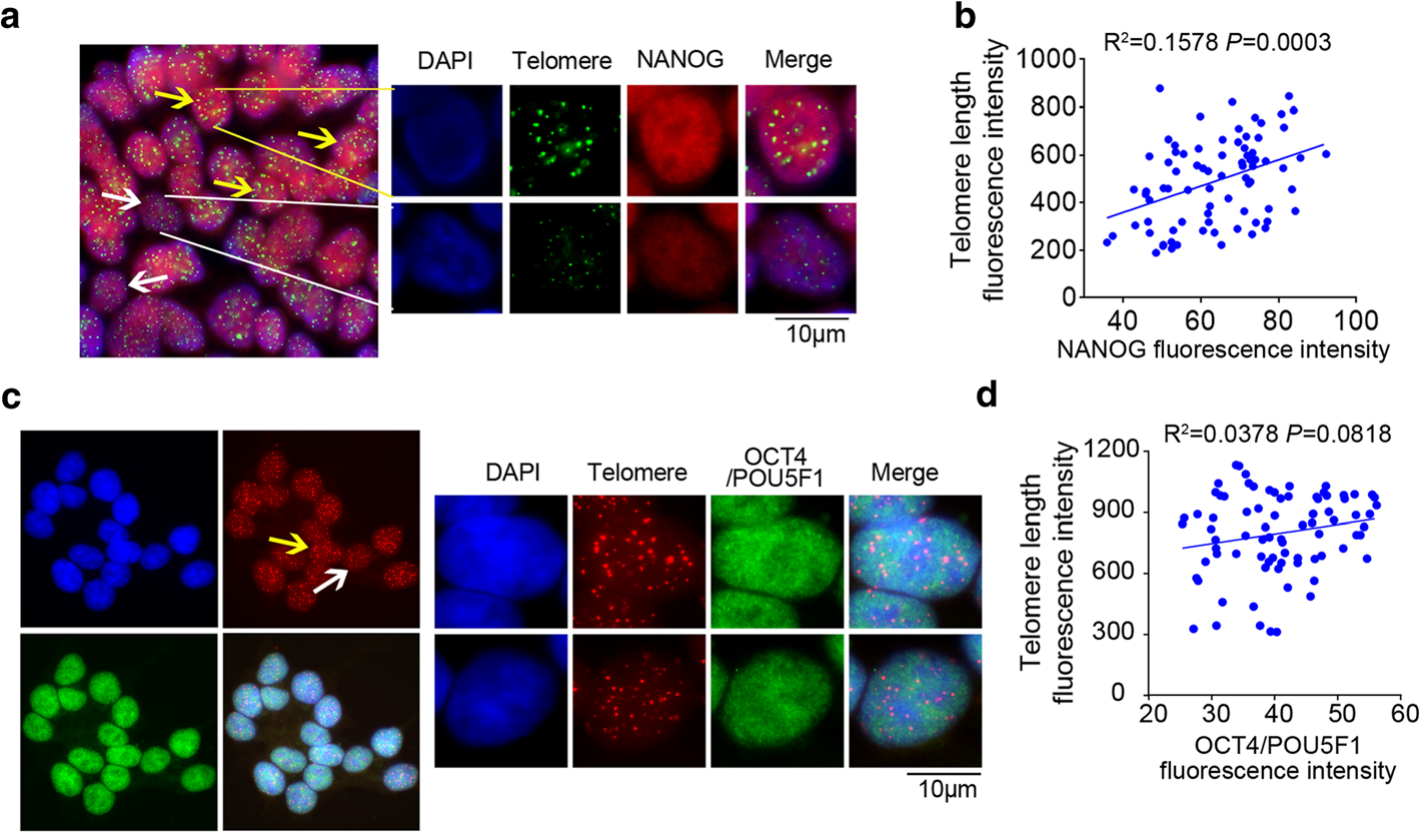
NANOG and OCT4/POU5F1 expression levels in association with telomere length and pluripotency of human embryonic stem cell (hESCs). **a** Telomere fluorescence signal (green, TelC-FITC, F1009, Panagene) and NANOG expression (red) in hESCs revealed by IF-FISH. DAPI-stained nuclei. **b** Linear regression analysis of telomere length and NANOG protein expression at single cell level. **c** Telomere fluorescence signal (red, TelC-Cy3, F1002, Panagene) and OCT4/POU5F1 expression (green) in hESCs revealed by IF-FISH. DAPI-stained nuclei. **d** Linear regression analysis of telomere length and OCT4/POU5F1 protein expression at single cell level

Immunofluorescence-FISH (IF-FISH) was employed to reveal NANOG expression and telomere length fluorescence signals in the same cell (Fig. 6a). The expression level of NANOG correlated well with telomere length (*P* < 0.01) (Fig. 6b). We also observed that expression levels of *NANOG* were positively correlated with telomere length in *TERC* ^-/-^ and WT hESCs at the single cell level using scT&R-seq (Additional file 2: Figure S2h). However, OCT4/POU5F1 expression showed much less heterogeneity in hESCs (Fig. 6c) and its expression level by the relative fluorescence intensity was minimally correlated with telomere length (Fig. 6d), further supporting RNA-seq data (Fig. 4e).

**Fig. 7:**
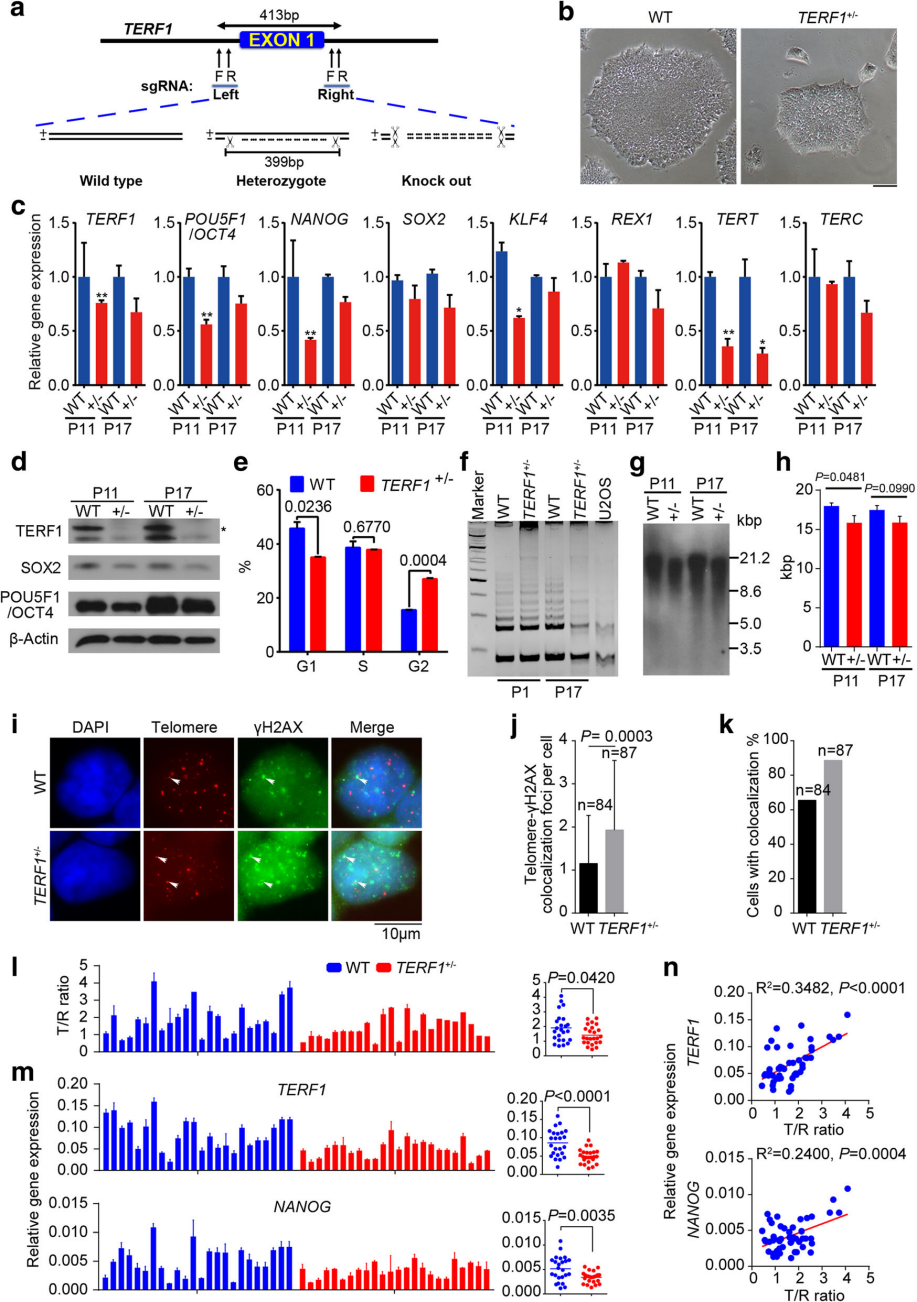
TERF1 expression levels associated with telomere length and pluripotency of human embryonic stem cell (hESCs). **a** Scheme for *hTERF1* knockout by CRISPR/Cas9. **b** Morphology of *TERF1* ^+/-^ and wild-type (WT) hESCs (WA26) at passage 11. Scale bar = 100 μm. **c** Expression levels of pluripotency and telomerase genes in *TERF1* ^+/-^ and WT hESCs by qPCR analysis. *P < 0.05, **P < 0.01, compared with WT controls. The data values of each gene are provided in Additional file 18: Table S10. **d** Western blot analysis of TERF1, SOX2, and OCT4/POU5F1 protein levels in *TERF1* ^+/-^ and WT hESCs. β-actin served as a loading control. **e** Cell cycle analysis of *TERF1* ^+/-^ and WT hESCs by flow cytometry. Data represent mean ± SD of three independent experiments. **f** Telomerase activity by TRAP assay of *TERF1* ^+/-^ and WT hESCs at different passages. U2OS served as a negative control. **g** Telomere length measurement by southern blot analysis of *TERF1* ^+/-^ and WT hESCs. **h** Quantification of southern blot results. Data represent mean ± SD of two independent experiments. **i** Immunofluorescence detection of γH2AX and telomere FISH of *TERF1* ^+/-^ and WT hESCs. **j**, **k** Number of telomere-γH2AX colocalization foci per cell (**j**) and percentage of cells with the colocalization (**k**). **l** Relative telomere length of individual *TERF1* ^+/-^ and WT hESCs by scT&R-seq. Right panel shows the mean telomere length by scT&R-seq. **m** Gene expression measured in single cells of *TERF1* ^+/-^ and WT hESCs by scT&R-seq. Right panel shows the mean level of gene expression. **n** Linear regression analysis of telomere length and gene expression (*TERF1* and *NANOG*) at single cell level

*TERF1* was highly expressed in hESCs with longer telomeres. To understand the role of *TERF1* in telomere length regulation and pluripotency of hESCs, we performed *TERF1* knock-out experiments in hESCs by CRISPR/Cas9. Homozygous *TERF1* ^-/-^ cell lines were not achievable by repeated experiments, suggesting that complete knockout of *TERF1* in hESCs might be lethal. Heterozygous *TERF1* ^+/-^ indicated deletion of 399 bp covering the whole exon1 of *TERF1* (Fig. 7a). Compared to WT hESCs, *TERF1* ^+/-^ hESCs showed smaller clones, reduced level of TERF1 and down-regulation of pluripotency genes, including *POU5F1*/*OCT4*, *NANOG*, *SOX2*, *KLF4*, and *REX1*/*ZFP42*, and of telomerase genes *TERT* and *TERC* (Fig. 7b–d), suggesting that TERF1 is important for pluripotency maintenance of hESCs. In addition, *TERF1* ^+/-^ hESCs displayed an altered cell cycle and elongated G2/M phase (Fig. 7e), and decreased telomerase activity with increasing passages (Fig. 7f). Telomeres were slightly shortened in *TERF1* ^+/-^ hESCs, compared with WT hESCs (Fig. 7g, h). It is unclear whether telomeres altered more in *TERF1* ^-/-^ hESCs, as the complete *TERF1* ^-/-^ knockout cell line was not achieved. It also will be necessary to assess whether *TERF1* functions differently in human ESCs in comparison with other somatic cell types. *Terf1* deficiency could increase telomere fragility and cancer in mice [50]. We showed that γH2AX foci were increased in telomeres in *TERF1* ^+/-^ cells (Fig. 7i–k), supporting the notion that telomeres of *TERF1* defective cells are prone to breakage [50]. Additionally, we analyzed the influence of *TERF1* deficiency on telomeres at single cell level using scT&R-seq. As a result, telomeres were shorter in *TERF1* ^+/-^ cells compared with WT hESCs (Fig. 7l), largely in agreement with TRF telomere distribution data (Fig. 7g, h). Moreover, expression levels of *TERF1* and *NANOG* were reduced in *TERF1* ^+/-^ hESCs (Fig. 7m), consistent with the bulk results (Fig. 7c). Expression levels of *NANOG* and *TERF1* were also positively correlated with telomere lengths at the single cell level measured by scT&R-seq (Fig. 7n). Other causes, aside from the reduced telomerase and shortened telomeres, might also drive gene expression changes in these cells.

## Discussion

Using simultaneous measurement of both transcriptome and telomere length in the same cell, we observed that telomere length heterogeneity links to pluripotency and, interestingly, to metabolism signaling representing a pluripotent state in human ESCs.

High expression levels of *NOP10* and *DKC1* may suggest their potential role in telomere maintenance of hESCs, presumably through the activities of the telomerase enzyme complex with the shelterin complex (*TERF1*, *TRF2*, *TINF2*, *POT1*, *TPP1*, and *RAP1*) [51]. ESCs with short telomeres have lower pluripotency, and pluripotent cells show more gene copy number variation than differentiation cells [52]. In addition to the genes already known for telomere length maintenance [53], our data reveals 16 candidate genes potentially associated with telomere lengths in hESCs (Additional file 7: Table S3). For instance, *MELK* and *MSH6* were expressed at higher levels in long telomere hESCs relative to those of short telomere cells. *MELK* is expressed in the mouse egg and preimplantation embryo [54] and may regulate proliferation of cancer stem cells [55]. *MSH6* is required for repair of mismatched DNA bases to avoid mutation and repress homologous recombination [56]. *MSH6* also is involved in NHEJ by association with Ku70/80 in DSB repair [57], and Ku70/80 interacts with both the protein and RNA components of human telomerase involved in telomere maintenance [58]. Further experiments are required to assess whether these genes are involved in telomere length regulation of hESCs.

Simultaneous measurement of telomere and transcriptome in the same cell reveals that long telomeres are linked to high expression of pluripotency marker genes of hESCs. This is of particular significance given that developmental pluripotency of hESCs could not be functionally tested by the methods that can be applied to mouse ESCs, including germline chimera tests or tetraploid embryo complementation. hESC pluripotent marker genes, such as *ZFP42* (*REX1*), *PRDM14*, and *NANOG* [59], are expressed at lower levels in short telomere ESCs than those of long telomere hESCs. Moreover, IF-FISH confirms higher NANOG expression level in long telomere hESCs than in short telomere cells, consistent with the notion that telomere lengths could be used to mark the pluripotency state of human ESCs. In addition, CRISPR/Cas9-mediated genome editing of *TERF1* suggests that *TERF1* plays an important role in hESCs, as *TERF1* ^-/-^ cells could be lethal, and pluripotency-related genes are down-regulated in *TERF1* ^+/-^ hESCs.

Excitingly, telomere length is implicated in metabolism signaling. Long telomere hESCs display higher glycolysis metabolic signaling than do short telomere cells. Primed and naïve hESCs exhibit a different metabolic state [17, 49, 60], and naïve hESCs show increased glycolysis compared to primed counterparts [49]. We find that genes for glycolysis metabolism, including *ENO3*, *SLC16A1*, *LDHA*, and *PFKM*, are expressed at higher levels in hESCs with long rather than short telomeres, and these are reportedly highly expressed in naïve hESCs relative to primed hESCs [49]. In contrast, genes for oxidative phosphorylation are highly expressed in short telomere cells. These data indicate that hESCs with long telomeres might maintain pluripotency by high glycolytic metabolism, whereas short telomeres may signal a tendency to differentiation by increased oxidative phosphorylation [49, 60]. Together, telomere lengths can indicate the pluripotency state of hESCs.

## Conclusions

Our analysis focused on heterogeneity in telomere length and pluripotency of hESCs. It has been recognized that tumors are more heterogeneous in their gene expression and telomere lengths than those of normal cells. We anticipate that the method of simultaneous analysis of telomere length and global transcription in the same cell will be useful in the further understanding of the molecular signaling underlying tumor heterogeneity and in the discovery of new targets for cancer treatment.

## Methods

### Cell culture

WA26 and RuES2 hESCs were routinely maintained in the undifferentiated state in E8 medium (A1517001, Life technologies) on Matrigel-coated (356230, BD Bioscience) tissue culture plates with daily medium change and passaged every 3 days using 0.5 mM EDTA in PBS at a 1:20 ratio with Rocki (sc-281642A, Santa Cruz). The colon carcinoma cell line (HCT116) was cultured at 37 °C in 5% CO_2_ in RPMI1640 (11875085, Life technologies) plus 10% FBS. Osteosarcoma cells (U2OS, ALT positive cell line), cervix adenocarcinoma cells (HeLa), and HEF cells were cultured in high glucose DMEM plus 10% FBS with 1% penicillin and streptomycin.

### Single-cell isolation and lysis

hESCs were separated following digestion with 0.5 mM EDTA in PBS and other cell types were separated following treatment with 0.25% Trypsin-EDTA. Single cells were resuspended in PBS with 0.1% BSA (A3311-10g, SIGMA), picked up in 1 μL 0.1% BSA using a micropipette with an epT.I.P.S. pipette tip (0030000838, Eppendorf) under a dissecting microscope, and transferred to the bottom of a 200-μL PCR tube (8-strip, nuclease-free, thin-walled PCR tubes with caps, PCR-0208-C, Axygen) consisting of 4 μL lysis buffer containing 3.45 μL of Buffer RLT plus (1053393, Qiagen), 0.5 μL Biotin-oligo-dT primer (5′-biotin-TEG-AAGCAGTGGTATCAACGCAGAGTACT_30_VN-3′), and 0.05 μL of Recombinant RNase inhibitor (2313A, Clontech).

### Separation of the genome and transcriptome in the same cell

Separation of gDNA and mRNA was performed as described previously [35]. Briefly, samples were incubated at 72 °C for 3 min (in our preliminary experiments, we found that this hybridization step could increase the efficiency of mRNA capture and bring better gene body coverage profile from 5′ to 3′), followed by hybridization of the Biotin-oligo-dT primer to poly(A)^+^ tail mRNA. Dynabeads in 4 μL (65001, Life technologies) were added to capture poly(A)^+^ tail mRNA, collected by a magnet, and the gDNA supernatant was transferred to a fresh PCR tube. Single-cell cDNA was synthesized in the tube containing mRNA, based on Smart-seq2 protocol [61]. The gDNA was purified by 8 μL Agencourt AMPure beads (A63881, Beckman Coulter). After washing with 80% (vol/vol) ethanol, the gDNA beads were freshly used or stored at −80 °C.

### Library construction and sequencing

The libraries were prepared by using TruePrep DNA Library Prep Kit V2 for Illumina® (TD503-02, Vazyme Biotech) according to the instruction manual. Samples were barcoded during library preparation and multiplex sequenced, with a 50-bp single-end sequencing strategy on a HiSeq 2500 (Illumina) in fast mode.

### Single-cell RNA-seq data analysis

Reads were mapped to hg19 from iGenome (https://support.illumina.com/sequencing/sequencing_software/igenome.html) by TopHat (v2.1.0) [62] with default parameters. Read counts of each gene annotated in RefGene were calculated by HTSeq with default parameters. Raw counts were normalized by library size via ‘*estimateSizeFactorsForMatrix*’ function from R package ‘*DESeq’*. High variable genes were identified based on the method developed by Brennecke et al. [63]. The squared coefficient of variation (CV^2^) and average count of each gene was calculated and fitted to a curve with the parameterization of CV^2^ = a1/μ + α0.

The gene counts were loaded into the R package *scater* and standard quality control metrics were calculated [40]. The package provides a convenient, flexible workflow to process raw sequencing reads into a high-quality expression dataset ready for downstream analysis.

PCA was conducted using log2 transformed TPM values, the PCA_int() function in R and the first two principal components of variance. The PluriNet score of single cells was calculated by averaging expression levels of PluriNet gene set [44]. Differential expression and function enrichment of genes between the putative groups were conducted using the R package SCDE [64].

### Analysis of cell cycle phase in association with gene expression profile

We examined the effect of the cell cycle on our clustering results by scLVM [38] and using cell cycle-related genes, including a previously defined core cell cycle gene set of cell cycle phase [65].

### Single-cell telomere length assay

Single-cell telomere length was measured using single-cell telomere length measurement by qPCR assay, as previously described [36]. A multiplex pre-amplification (pre-PCR) step that can amplify telomere repeats (T) and *Alu* reference gene (R) were simultaneously employed with telomere primers (forward primer: CGGTTTGTTTGGGTTTGGGTTTGGGTTTGGGTTTGGGTT, reverse primer: GGCTTGCCTTACCCTTACCCTTACCCTTACCCTTACCCT), and *Alu* primers (forward primer: GACCATCCCGGCTAAAACG, reverse primer: CGGGTTCACGCCATTCTC). The reactions were set up by 25-μL aliquota of a master mix with single-cell gDNA beads. Each reaction was set up with 2.5 μL of 10X iTaq buffer, 1.5 mM MgCl_2_, 0.625U iTaq DNA polymerase (170-8870, Bio-Rad), 0.5 μL of 10 mM dNTP mix (170-8874, Bio-Rad), 1 μL each of telomere forward and reverse primers (10 μM), and 1 μL each of *Alu* forward and reverse primers (10 μM). Thermal cycler reaction conditions were set at 95 °C for 3 min followed by 18 cycles of 95 °C for 30 s, 60 °C annealing for 30 s, and extension at 72 °C for 30 s. PCR products were purified using DNA clean and concentrator-5 kit (D4004, Zymo Research) and eluted in 64 μL of double distilled water. iQ™ SYBR® Green-based real-time PCR (170-8882, Bio-Rad) was performed using the same primer sets. Relative telomere length (T/R ratio) was calculated by comparing the values of telomere (T) and reference gene *Alu* (R) in individual cells by the 2^-ΔΔCt^ method when the standard curves of telomere and *Alu* showed similar high amplification efficiency. We defined the cells with T/R ratio over 1.0 as the “long” telomere group, T/R ratio ranges from 0.4 to 1.0 as the “medium” group, and T/R ratio under 0.4 as the “short” group under our experimental conditions.

### Telomere measurement by quantitative real-time PCR (T/S ratio)

Genomic DNA was extracted using DNeasy Blood & Tissue kit (69504, Qiagen) and the ratio of 260 to 280 was between 1.8 and 2.1. Average telomere length was measured using qPCR assay, as previously described [27, 36]. Each 20 μL reaction was performed as follows: 35 ng gDNA, 1 × SYBR Green master mix (QPK-201, TOYOBO), 250 nM telomere forward primer and 250 nM telomere reverse primer, or *36B4* primers (forward primer: CAGCAAGTGGGAAGGTGTAATCC, reverse primer: CCCATTCTATCATCAACGGGTACAA). The telomere signal (T) was normalized to the signal from single copy gene (S) human *36B4* to generate a T/S ratio indicative of relative telomere length according to a standard curve. Three repeat reactions were performed for each sample.

### Telomere terminal restriction fragment (TRF) by Southern blot analysis

TRF analysis was performed as described using TeloTAGGG Telomere Length Assay Kit (12209136001, Roche). DNA (1.5 μg) was digested using *Hinf* I and *Rsa* I restriction enzymes. Digested DNA underwent electrophoresis through a 0.8% agarose gel (111860, Biowest) for 4 h at 6 V/cm in 1× TAE buffer. Gels were denatured, neutralized, and transferred to positively charged nylon membranes (RPN2020B, GE Healthcare). The membranes were hybridized in DIG Easy Hyb containing telomere probe at 42 °C overnight. Mean TRF length was quantitatively measured according to the kit instructions.

### Single-cell qPCR

Single-cell mRNA was directly reverse transcribed to cDNA by Smart-seq2, and the product diluted at 0.25 ng/μL. Real-time qPCR reaction was performed using FastStart Universal SYBR Green Master (4913914001, Roche). All genes were confirmed for their specificity by dissociation curves and amplification curves, and *GAPDH* served as housekeeping gene for normalization of gene expression. Primers used for qPCR experiments are listed in Additional file 19: Table S11.

### Immunofluorescence microscopy and quantification

Cells were washed twice with PBS, then fixed in freshly prepared 3.7% paraformaldehyde for 15 min at 4 °C, permeabilized with 0.1% Triton X-100 in blocking solution (3% goat serum plus 0.5% BSA in PBS) for 40 min at room temperature, washed three times (each for 15 min), and left in blocking solution for 1 h. Cells were incubated overnight at 4 °C with primary antibodies against OCT4/POU5F1 (Cat# sc-9081, Lot# G0607, RRID: AB_2167703, Santa Cruz), NANOG (Cat# sc-293121, Lot# F2716, RRID:AB_2665475, Santa Cruz), and γH2AX (Cat# ab11175, RRID:AB_297814) and incubated for 1 h with fluorescence-labeled secondary antibodies. Samples were washed and counterstained with 0.5 μg/mL DAPI in Vectashield mounting medium. Fluorescence was detected and imaged using a Zeiss inverted fluorescence microscope. IF-FISH was performed as described [66]. Integrated fluorescence intensity was estimated using ImageJ software, and the threshold was defined using non-specific background staining fluorescence.

### Knockout of *TERF1* or *TERC* by CRISPR/Cas9

pSpCas9(BB)-2A-Puro (PX459, Addgene plasmid #48139) and pSpCas9(BB)-2A-GFP (PX458, Addgene plasmid #48138) were a gift from Feng Zhang. Guide RNAs were designed using the online design tool available at http://crispr.genome-engineering.org/. PX458/PX459 was digested with *Bbs*I and then gel purified. Two pairs of oligos including targeting sequences were annealed, guide RNAs of *TERF1* were cloned into *Bbs*I-digested PX459, and guide RNAs of *TERC* were cloned into *Bbs*I-digested PX458. Primers used for CRISPR/Cas9 experiments are listed in Additional file 19: Table S11.

### Western blot

Western blot was performed as described previously [66] and the antibodies used were TRF1/TERF1 (Cat# ab10579, Lot# GR253870-4, RRID:AB_2201461, Abcam), OCT4/POU5F1 (Cat# sc-9081, Lot# G0607, RRID: AB_2167703, Santa Cruz), SOX2 (Cat# AB5603, Lot# 2762353, RRID: AB_2286686, Millipore), and β-actin (Cat# sc-1616R, Lot# A3009, RRID: AB_630836, Santa Cruz). The protein bands were detected by Enhanced ECL Amersham^TM^ prime Western blotting detection reagent (RPN2232, GE Healthcare).

### Telomerase activity assay

Telomerase activity was determined using TeloChaser Telomerase assay kit (TLK-101, TOYOBO). Approximately 2.5 × 10^4^ cells from each sample were lysed, and heated at 70 °C for 10 min to serve as negative controls. PCR products of cell lysates were separated on non-denaturing TBE-based 12% polyacrylamide gel electrophoresis and visualized by ethidium bromide staining.

### Gene expression by quantitative real-time PCR

Total RNA was isolated from cells using RNeasy mini kit (74104, Qiagen). RNA (2 μg) was subjected to cDNA synthesis using M-MLV Reverse Transcriptase (28025021, Invitrogen). Real-time quantitative PCR reactions were set up in duplicate with FastStart Universal SYBR Green Master (4913914001, Roche) and run on the iCycler iQ5 2.0 Standard Edition Optical System (Bio-Rad). Each sample was repeated three times and analyzed using *GAPDH* as the internal control. Primers used for qPCR experiments are listed in Additional file 19: Table S11.

### Cell cycle analysis

Cells were fixed in freshly prepared 70% ethanol at 4 °C overnight, then centrifuged at 1000 *g* for 5 min and stained with propidium iodide at 37 °C for 30 min in a water bath. FACS analysis was used to determine cell cycle phases.

### Statistics and reproducibility

Correlation between telomere length and expression level of genes was analyzed using Pearson’s correlation. Significance in different groups was analyzed by Student’s *t* test (two-paired groups) and ANOVA (multiple groups). Significant differences were defined as *P < 0.05 and **P < 0.01. The results were shown as mean ± SD.

## Additional files

Additional file 1: Figure S1. Measurement of relative telomere length in single cells by scT&R-seq of various human cell lines and validation by other established methods. To validate the feasibility of this method, we analyzed the telomere length and gene expression of six single cultured osteosarcoma cells (U2OS, ALT pathway), and six single human ESCs (WA26, telomerase activity-positive). (a) Mean Ct value of single human cell after preamplification by telomere and *Alu* primers in WA26 and U2OS. Both Ct value of *Tel* and *Alu* of negative controls are much greater than those of single cells. (b) Standard curves used for calculation of relative telomere length by scT&R-seq. Standard curves of telomeres and *Alu* using human embryonic fibroblasts (HEF) show remarkable linear correlation from DNA concentrations 0.2 ng to 125 ng, indicating a high amplification efficiency of telomeres and *Alu*. (c) Mean Ct value of *GAPDH* from the same cell by scT&R-seq and value of *GAPDH* does not differ in these cells. *OCT4*/*POU5F1* is highly expressed in hESC but not in U2OS, so WA26 has a low Ct value for *POU5F1*. (d) Comparison of telomere length by the method without separation step and scT&R-seq method. (e) Relative telomere length of individual cells from human ESC WA26 and RuES2, human cancer cells U2OS, HCT116 and HeLa, and HEF. (f) Telomere length measurement by scT&R-seq. (g) Telomere length measurement by qPCR (T/S ratio). (h) Measurement of telomere length by southern blot of terminal restriction fragments (TRF). (i) Immunofluorescence images of OCT4/POU5F1 protein expression level in WA26 and HEF cells. Scale bar = 50 μm. (j) TRAP assay of telomerase activity in hESC (WA26) and U2OS. IC, internal control; HI, heat-inactivated; NT, not treated. All values indicate mean ± SD from three independent experiments. (PDF 888 kb)

Additional file 2: Figure S2. Validation of telomere length and gene expression by scT&R-seq of *TERC* knockout and WT hESCs. (a) Morphology of *TERC* ^-/-^ and WT hESCs. Scale bar = 200 μm. Two pairs of guide RNAs were cloned into pSpCas9(BB)-2A-GFP (PX458, Addgene plasmid # 48138, a gift from Feng Zhang) for construction of the *TERC* knockout cells. Primers used for CRISPR/Cas9 experiments are listed in Additional file 19: Table S11. (b) Telomerase activity by TRAP assay of *TERC* ^-/-^ and WT hESCs. (c) Telomere length by TRF of hESCs after *TERC* knockout. (d) Relative telomere length of single cells from *TERC* ^-/-^ and WT hESCs using scT&R-seq. (e) Comparison of Ct values for housekeeping gene (*GAPDH*) in *TERC* ^-/-^ and WT hESCs. (f) Single cell qPCR analysis of expression of *NANOG* in the same cell from *TERC* ^-/-^ and WT hESCs using scT&R-seq. (g) Relative expression levels by qPCR of *NANOG* in bulk *TERC* ^-/-^ and WT hESCs. (h) Linear regression analysis of telomere length and *NANOG* expression at single cell level. (PDF 590 kb)

Additional file 3: Table S1. Single cell telomere length T/R ratio and PluriNet score. Telomere length of single hESCs by scT&R-seq and PluriNet score of single cell by average of the expression level of genes from PluriNet gene set. (XLSX 16 kb)

Additional file 4: Figure S3. Quality control of single-cell RNA-seq analysis. (a) Cumulative gene diversity and gene-body coverage profile across lengths of all genes, and the reads coverage along the position from 5′ to 3′. (b, c) Total mapped reads and mapped ratio of single cell RNA-seq data. The reads were filtered and mapped to hg19 (ftp://igenome:G3nom3s4u@ussd-ftp.illumina.com/Homo_sapiens/UCSC/hg19/Homo_sapiens_UCSC_hg19.tar.gz) by TopHat (v2.1.0) with default parameters. Read counts for each gene were calculated for each replicate using HTSeq with default parameters. (d) Total number of genes detected with different transcripts per million value (TPM). (e) Heat-map shows top 40 genes that are highly expressed in all hESCs. The genes are ranked based on average of TPM value of all cells. (f) Plot of gene expression profile related to cell-cycle phases of hESCs, and distribution of telomere length at various cell cycle phases. (PDF 1307 kb)

Additional file 5: Table S2. Normalized counts (TPM) for all 121 single cells. All detected genes’ (>10 counts in ≥ 5 single cells) raw counts were normalized by library size via ‘*estimateSizeFactorsForMatrix*’ function from R package *DESeq*. (XLSX 15470 kb)

Additional file 6: Figure S4. Validation of single-cell RNA-seq data by quantitative real-time PCR. (a, c) Single cell gene expression profile revealed by single cell RNA-seq. (b, d) Relative single cell gene expression level by single cell qPCR. (e, f) Correlation between RNA-seq and qPCR evaluated by Pearson’s correlation coefficient. (PDF 416 kb)

Additional file 7: Table S3. Top genes in the expression levels showing positive or negative association with telomere length of hESCs. (XLSX 9 kb)

Additional file 8: Table S4. Telomere length in association with expression level of all detected genes. (XLSX 588 kb)

Additional file 9: Table S5. Results of principal component analysis (PCA). All PCAs presented in this manuscript were conducted using log2 (TPM + 1) values of all detected genes using the PCA_int() function. (XLS 178 kb)

Additional file 10: Figure S5. The mapping ratio of single cell libraries is not correlated with telomere length (T/R ratio). (a) Cumulative gene diversity plot showing the proportion of the library size accounted for by the expressed features across different telomere groups. (b) Linear regression analysis of telomere length (T/R ratio) and mapping ratio at single cell level. (PDF 223 kb)

Additional file 11: Table S7. Differential gene expression in various groups based on T/R ratio and PluriNet score. (XLSX 307 kb)

Additional file 12: Figure S6. Functional annotation analysis of the differentially expressed genes from long, medium and short telomere groups. Functional annotation was performed by the assignment of Gene Ontology (GO) terms, and differentially expressed genes were annotated based on the Kyoto Encyclopedia of Genes and Genomes (KEGG) using the R package SCDE. (PDF 1040 kb)

Additional file 13: Table S6. Complete list of gene sets used in this study, including selected housekeeping, pluripotency and differentiation genes, and genes for PluriNet and telomere length maintenance from GSEA. (XLSX 14 kb)

Additional file 14: Figure S7. PluriNet score analysis of pluripotency state of human ESCs and differentiated cells. (a) Verification of PluriNet score for characterization of the pluripotency level*.* We validated the pluripotency score (named PluriNet score) using the single cell data of human pluripotent stem cells and differentiation cells from Thomson’s group [45]. PluriNet score displayed higher in undifferentiated hESCs than did differentiated cells from the same precursor cell. (b) Heat-map shows expression of PluriNet genes in all hESCs. Gene list of PluriNet was obtained from the GSEA website (Additional file 14: Table S6). The vertical color scale is shown as log(TPM + 1). (PDF 4032 kb)

Additional file 15: Table S8. GO Biological Process enrichment for up-regulated genes based on T/R ratio and PluriNet score group by SCDE. (XLSX 51 kb)

Additional file 16: Figure S8. Heat-map showing expression of marker genes for human naïve ESCs. Of the 12 selected genes related to pluripotency, genes for naïve pluripotency, *NANOG*, *KLF2*, *KLF4*, *TBX3*, *DPPA3* (*STELLA*), *DPPA5*, *PRDM14*, *ESRRB*, and *REX1* are expressed at high levels in human naïve ESCs, whereas *OTX2*, *ZIC2*, *ZIC3*, and *FGF5* decreased in human naïve ESCs. The vertical color scale is shown as log(TPM + 1). (PDF 1132 kb)

Additional file 17: Table S9. Enriched KEGG pathways for down-regulated genes in long vs. short telomere groups. (XLS 4 kb)

Additional file 18: Table S10. Quantification of gene expression by RT-qPCR. Gene expression levels were calculated by 2^-ΔΔCt^ method, *GAPDH* served as an internal control. (XLSX 10 kb)

Additional file 19: Table S11. Primer sequences. Primers for knock-out of *hTERF1* and *hTERC* by CRISPR/Cas9, and primers for qPCR. (XLSX 10 kb)

## Abbreviations

ALT: alternative lengthening of telomeres
ESC: embryonic stem cell
GO: Gene ontology
HEF: human embryonic fibroblast cell
IF-FISH: immunofluorescence-fluorescence in situ hybridization
iPSC: induced pluripotent stem cell
KEGG: Kyoto Encyclopedia of Genes and Genomes
PCA: principal component analysis
Q-FISH: quantitative fluorescence in situ hybridization
qPCR: real-time quantitative polymerase chain reaction
scT&R-seq: single cell telomere length and transcriptome sequencing
TPM: transcripts per million values
TRAP: telomeric repeat amplification protocol
TRF: telomere terminal restriction fragment
WT: wild-type
