# Supplementary figures and images for: Telomere heterogeneity linked to metabolism and pluripotency state revealed by simultaneous analysis of telomere length and RNA-seq in the same human embryonic stem cell
Source: BMC Biol. 2017 Dec 8;15:114. doi: 10.1186/s12915-017-0453-8 (PMC5721592; doi:10.1186/s12915-017-0453-8)

Figure S1

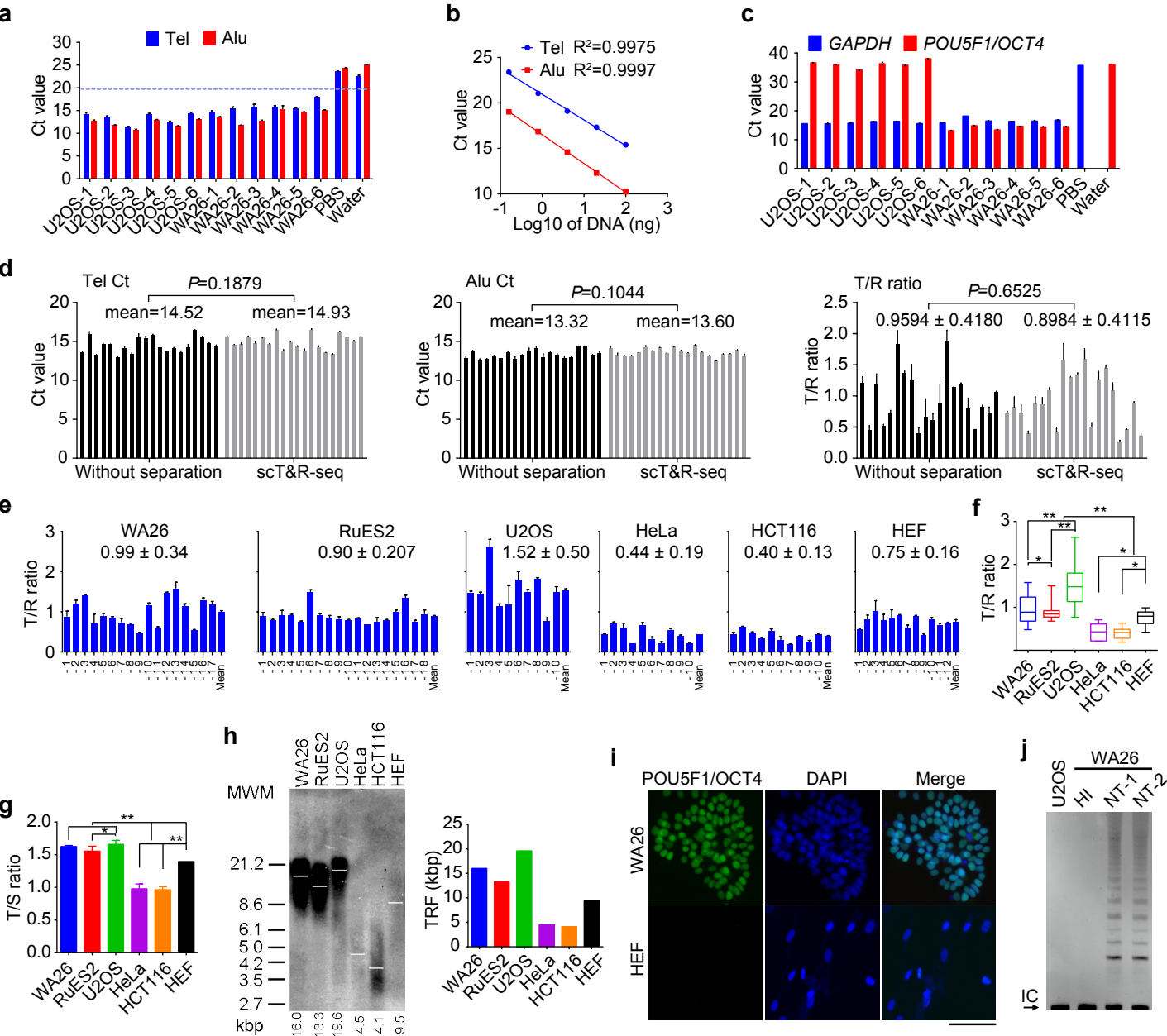

Supplement: Supplementary file 1 — Measurement of relative telomere length in single cells by scT&R-seq of various human cell lines and validation by other established methods. To validate the feasibility of this method, we analyzed the telomere length and gene expression of six single cultured osteosarcoma cells (U2OS, ALT pathway), and six single human ESCs (WA26, telomerase activity-positive). (a) Mean Ct value of single human cell after preamplification by telomere and Alu primers in WA26 and U2OS. Both Ct value of Tel and Alu of negative controls are much greater than those of single cells. (b) Standard curves used for calculation of relative telomere length by scT&R-seq. Standard curves of telomeres and Alu using human embryonic fibroblasts (HEF) show remarkable linear correlation from DNA concentrations 0.2 ng to 125 ng, indicating a high amplification efficiency of telomeres and Alu. (c) Mean Ct value of GAPDH from the same cell by scT&R-seq and value of GAPDH does not differ in these cells. OCT4/POU5F1 is highly expressed in hESC but not in U2OS, so WA26 has a low Ct value for POU5F1. (d) Comparison of telomere length by the method without separation step and scT&R-seq method. (e) Relative telomere length of individual cells from human ESC WA26 and RuES2, human cancer cells U2OS, HCT116 and HeLa, and HEF. (f) Telomere length measurement by scT&R-seq. (g) Telomere length measurement by qPCR (T/S ratio). (h) Measurement of telomere length by southern blot of terminal restriction fragments (TRF). (i) Immunofluorescence images of OCT4/POU5F1 protein expression level in WA26 and HEF cells. Scale bar = 50 μm. (j) TRAP assay of telomerase activity in hESC (WA26) and U2OS. IC, internal control; HI, heat-inactivated; NT, not treated. All values indicate mean ± SD from three independent experiments. (PDF 888 kb) [file 12915_2017_453_MOESM1_ESM.pdf]

Figure S2

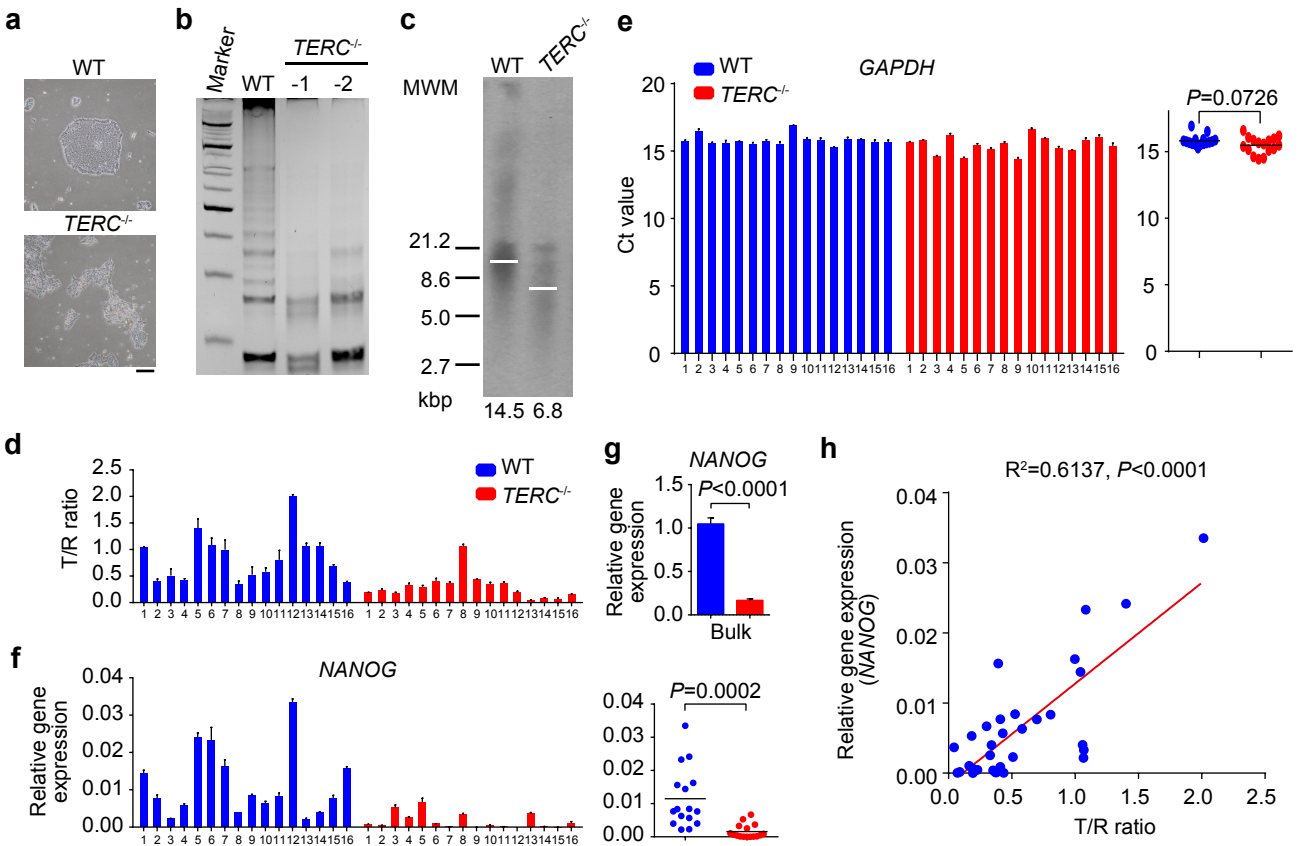

Supplement: Supplementary file 2 — Validation of telomere length and gene expression by scT&R-seq of TERC knockout and WT hESCs. (a) Morphology of TERC -/- and WT hESCs. Scale bar = 200 μm. Two pairs of guide RNAs were cloned into pSpCas9(BB)-2A-GFP (PX458, Addgene plasmid # 48138, a gift from Feng Zhang) for construction of the TERC knockout cells. Primers used for CRISPR/Cas9 experiments are listed in Additional file 19: Table S11. (b) Telomerase activity by TRAP assay of TERC -/- and WT hESCs. (c) Telomere length by TRF of hESCs after TERC knockout. (d) Relative telomere length of single cells from TERC -/- and WT hESCs using scT&R-seq. (e) Comparison of Ct values for housekeeping gene (GAPDH) in TERC -/- and WT hESCs. (f) Single cell qPCR analysis of expression of NANOG in the same cell from TERC -/- and WT hESCs using scT&R-seq. (g) Relative expression levels by qPCR of NANOG in bulk TERC -/- and WT hESCs. (h) Linear regression analysis of telomere length and NANOG expression at single cell level. (PDF 590 kb) [file 12915_2017_453_MOESM2_ESM.pdf]

Figure S3

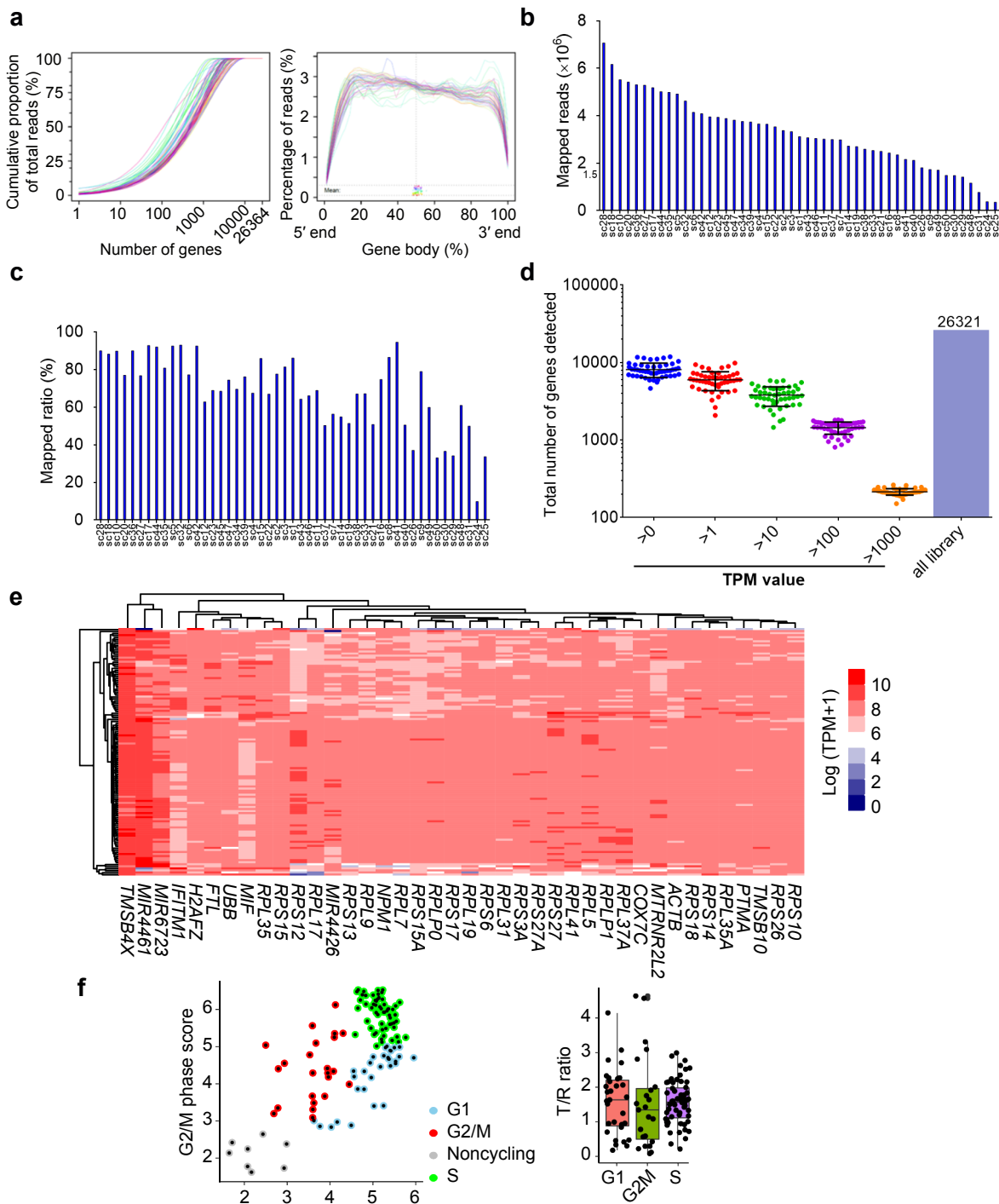

Supplement: Supplementary file 4 — Quality control of single-cell RNA-seq analysis. (a) Cumulative gene diversity and gene-body coverage profile across lengths of all genes, and the reads coverage along the position from 5′ to 3′. (b, c) Total mapped reads and mapped ratio of single cell RNA-seq data. The reads were filtered and mapped to hg19 (ftp://igenome:G3nom3s4u@ussd-ftp.illumina.com/Homo_sapiens/UCSC/hg19/Homo_sapiens_UCSC_hg19.tar.gz) by TopHat (v2.1.0) with default parameters. Read counts for each gene were calculated for each replicate using HTSeq with default parameters. (d) Total number of genes detected with different transcripts per million value (TPM). (e) Heat-map shows top 40 genes that are highly expressed in all hESCs. The genes are ranked based on average of TPM value of all cells. (f) Plot of gene expression profile related to cell-cycle phases of hESCs, and distribution of telomere length at various cell cycle phases. (PDF 1307 kb) [file 12915_2017_453_MOESM4_ESM.pdf]

Figure S4

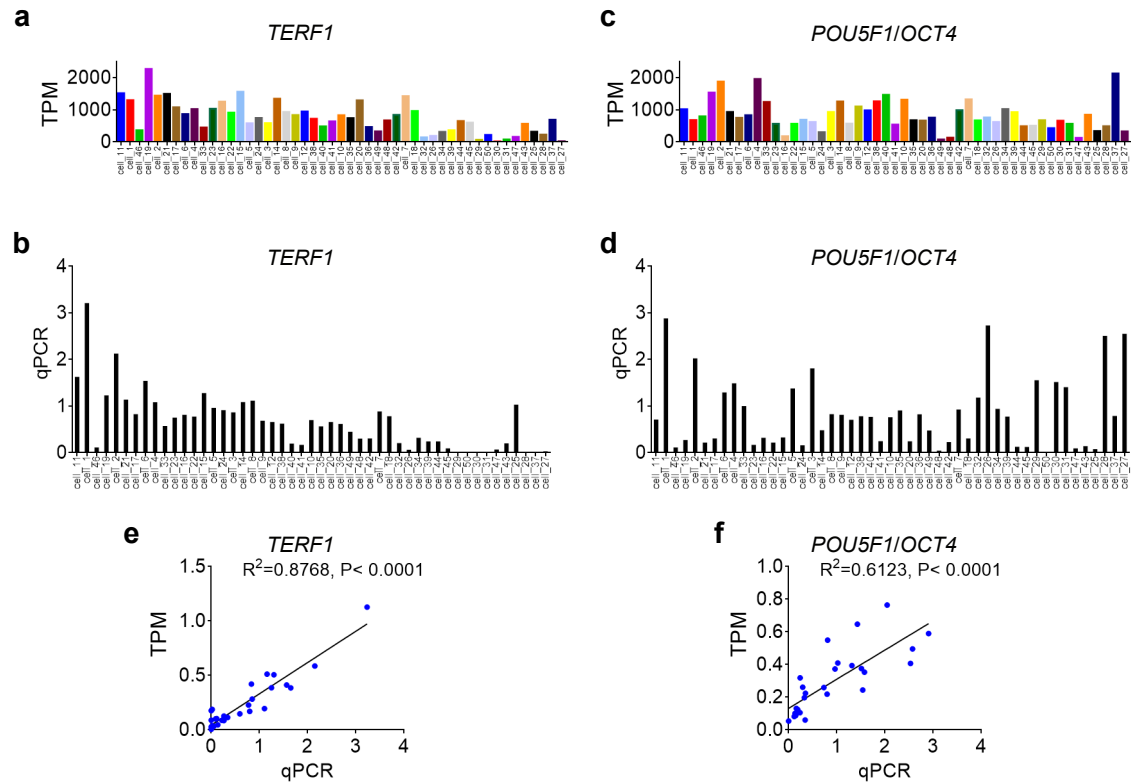

Supplement: Supplementary file 6 — Validation of single-cell RNA-seq data by quantitative real-time PCR. (a, c) Single cell gene expression profile revealed by single cell RNA-seq. (b, d) Relative single cell gene expression level by single cell qPCR. (e, f) Correlation between RNA-seq and qPCR evaluated by Pearson’s correlation coefficient. (PDF 416 kb) [file 12915_2017_453_MOESM6_ESM.pdf]

Figure S5

**a**

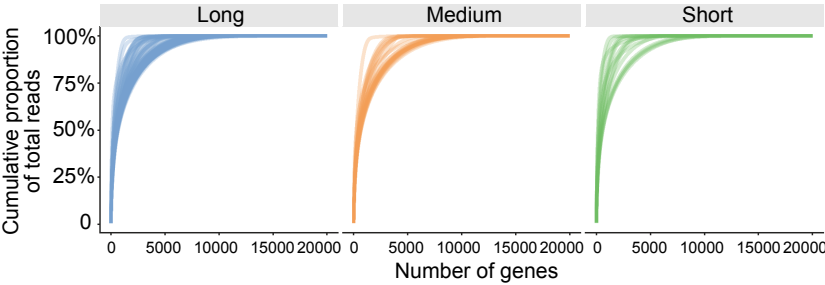

**b**

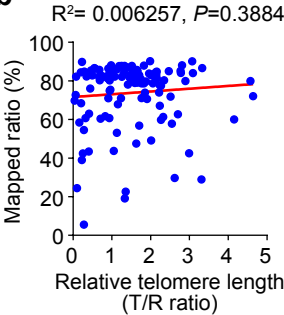

Supplement: Supplementary file 10 — The mapping ratio of single cell libraries is not correlated with telomere length (T/R ratio). (a) Cumulative gene diversity plot showing the proportion of the library size accounted for by the expressed features across different telomere groups. (b) Linear regression analysis of telomere length (T/R ratio) and mapping ratio at single cell level. (PDF 223 kb) [file 12915_2017_453_MOESM10_ESM.pdf]

Figure S6

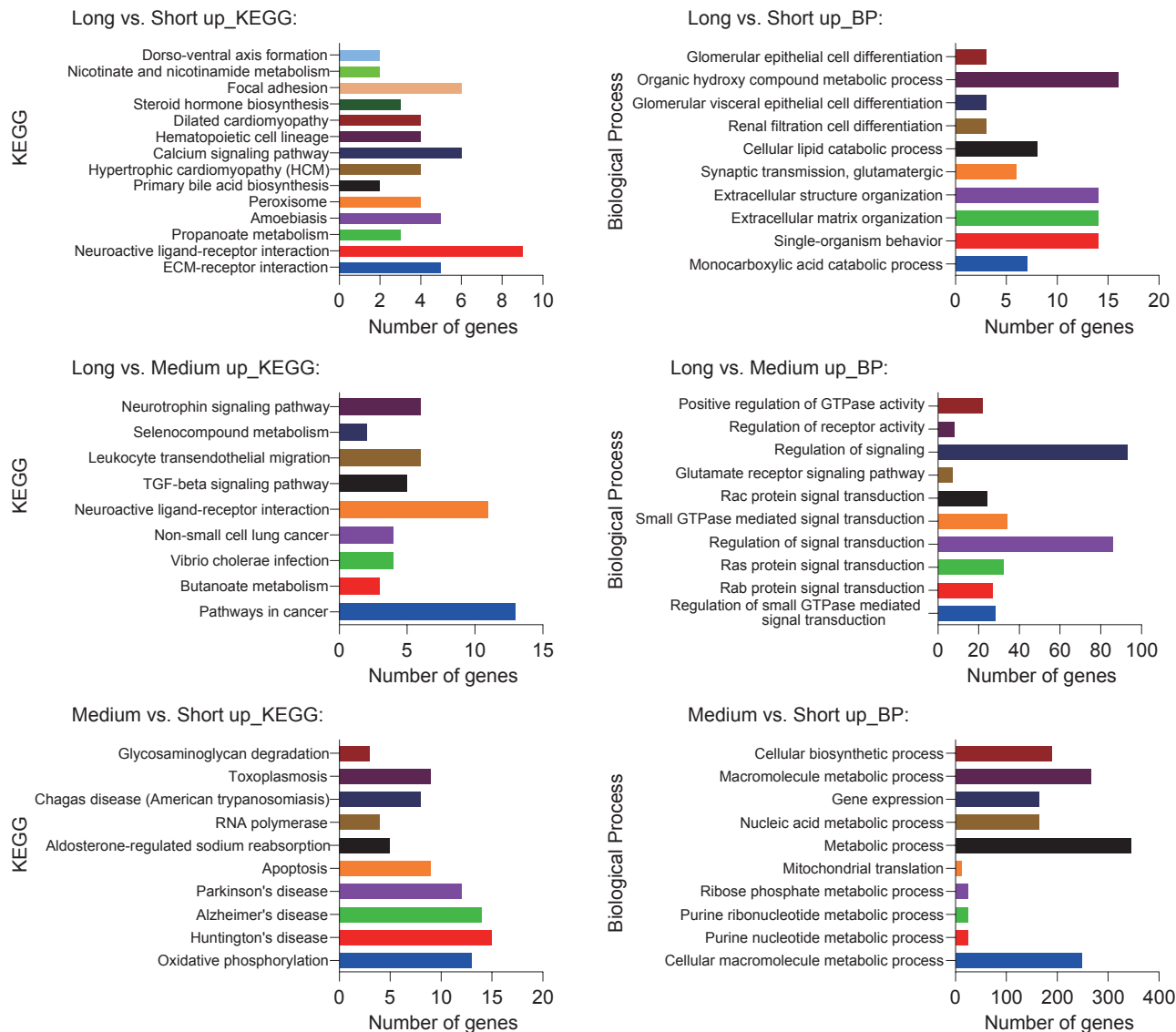

Supplement: Supplementary file 12 — Functional annotation analysis of the differentially expressed genes from long, medium and short telomere groups. Functional annotation was performed by the assignment of Gene Ontology (GO) terms, and differentially expressed genes were annotated based on the Kyoto Encyclopedia of Genes and Genomes (KEGG) using the R package SCDE. (PDF 1040 kb) [file 12915_2017_453_MOESM12_ESM.pdf]

Figure S7

**a**

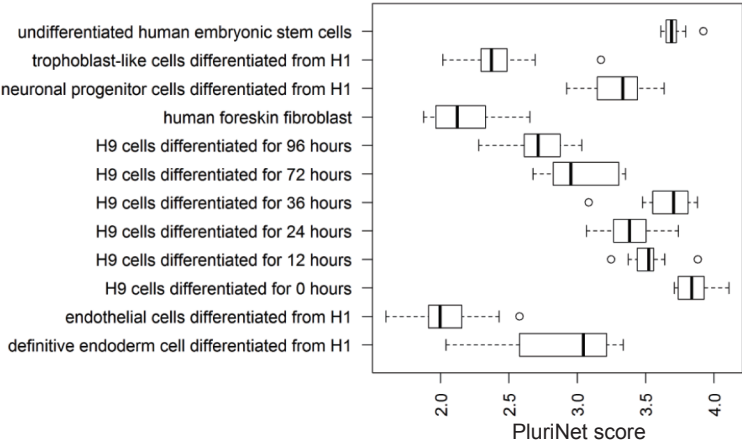

**b**

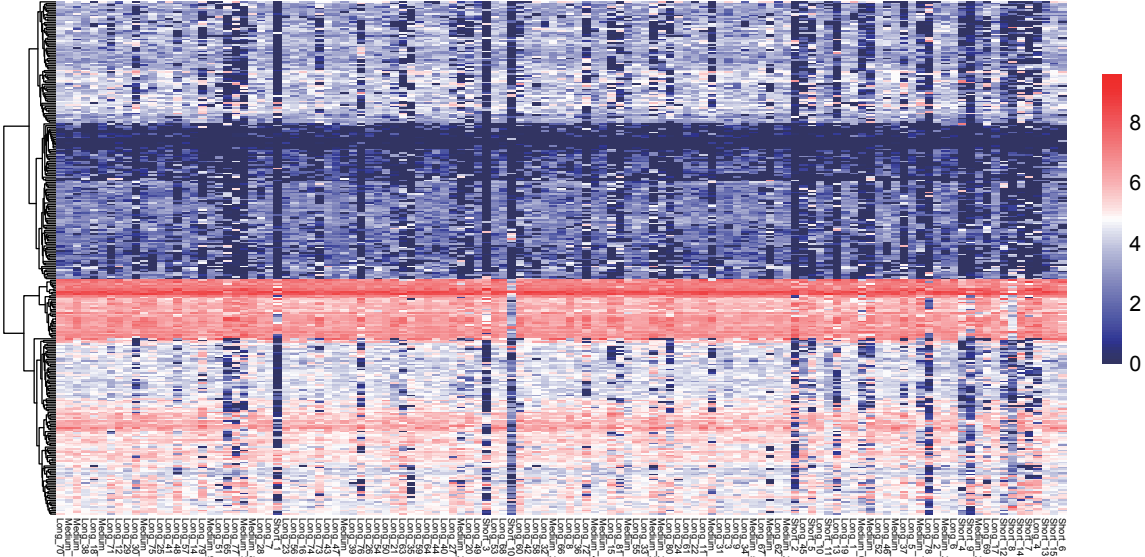

Supplement: Supplementary file 14 — PluriNet score analysis of pluripotency state of human ESCs and differentiated cells. (a) Verification of PluriNet score for characterization of the pluripotency level. We validated the pluripotency score (named PluriNet score) using the single cell data of human pluripotent stem cells and differentiation cells from Thomson’s group [45]. PluriNet score displayed higher in undifferentiated hESCs than did differentiated cells from the same precursor cell. (b) Heat-map shows expression of PluriNet genes in all hESCs. Gene list of PluriNet was obtained from the GSEA website (Additional file 14: Table S6). The vertical color scale is shown as log(TPM + 1). (PDF 4032 kb) [file 12915_2017_453_MOESM14_ESM.pdf]

suppl Figure 8

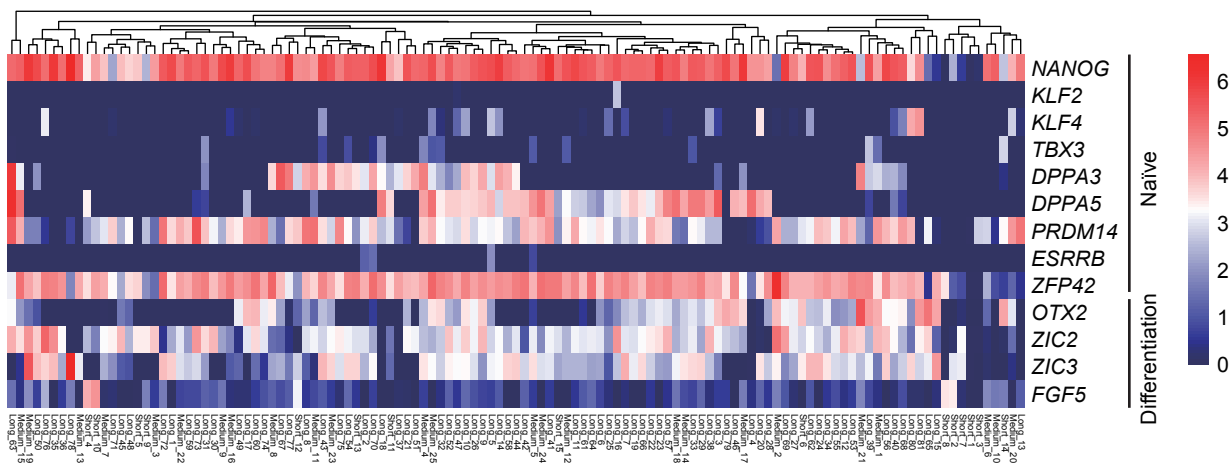

Supplement: Supplementary file 16 — Heat-map showing expression of marker genes for human naïve ESCs. Of the 12 selected genes related to pluripotency, genes for naïve pluripotency, NANOG, KLF2, KLF4, TBX3, DPPA3 (STELLA), DPPA5, PRDM14, ESRRB, and REX1 are expressed at high levels in human naïve ESCs, whereas OTX2, ZIC2, ZIC3, and FGF5 decreased in human naïve ESCs. The vertical color scale is shown as log(TPM + 1). (PDF 1132 kb) [file 12915_2017_453_MOESM16_ESM.pdf]
